# Scoping the proximal and distal dimensions of climate change on health and wellbeing

**DOI:** 10.1186/s12940-017-0329-y

**Published:** 2017-12-05

**Authors:** George Paterson Morris, Stefan Reis, Sheila Anne Beck, Lora Elderkin Fleming, William Neil Adger, Timothy Guy Benton, Michael Harold Depledge

**Affiliations:** 1 European Centre for Environment and Human Health, University of Exeter Medical School C/o Knowledge Spa RCHT, Truro, Cornwall, TR1 3HD UK; 2 NERC Centre for Ecology & Hydrology, Bush Estate, Midlothian, Penicuik UK; 3 NHS Health Scotland, Meridian Court, Cadogan Street, Glasgow, UK; 4 Geography, College of Life and Environmental Sciences, University of Exeter, Rennes Drive, Exeter, EX4 4RJ UK; 5 UK’s Global Food Security Programme and School of Biology, University of Leeds, Leeds, UK

**Keywords:** Ecosystems, Food, Nutrition, Mobility, Migration, Concepts, Stakeholder engagement, Theoretical frameworks, Ecosystem services

## Abstract

The impacts of climate on health and wellbeing occur in time and space and through a range of indirect, complicated mechanisms. This diversity of pathways has major implications for national public health planning and influence on interventions that might help to mitigate and adapt to rapidly changing environmental conditions, nationally and internationally. This paper draws upon evidence from public health and adverse impact studies across climate science, hydrology, agriculture, public health, and the social sciences. It presents a conceptual model to support decision-making by recognizing both the *proximal* and *distal* pathways from climate-induced environmental change to national health and wellbeing. The *proximal* and *distal* pathways associated with food security, migration and mobility illustrate the diverse climate change influences in different geographic locations over different timescales. We argue that greater realization and articulation of proximal and distal pathways should radically alter how climate change is addressed as a national and international public health challenge.

**Electronic supplementary material:**

The online version of this article (10.1186/s12940-017-0329-y) contains supplementary material, which is available to authorized users.

## Background

The effects of global climate change are now observable in every part of the world. Scientific assessments suggest that nowhere will be immune to the future threats climate change poses to human health and wellbeing [1]. Remarkably, many of the indirect adverse health impacts driven by climate-related ecological disruption and their consequences remain to be explored. Crop failures and shifting patterns in disease vectors are remote from current decision-making on energy systems and the aggregating emissions of greenhouse gases. Impacts emerge both from the physical and ecological changes across the globe, and from the societal responses such as geographic and social displacement of populations in conditions of prolonged drought or of severe and persistent flooding. Behaviors and lifestyles, as well as health, social and economic inequalities, will be profoundly affected by climate change [2, 3].

This paper does not seek to systematically review the health impacts of climate change but rather to reinforce the need for any country or community to better capture and communicate its true public health implications in a policy-relevant way. We focus here on the adverse impacts of climate change on health and wellbeing, through what we define as proximal and distal pathways. We adopt and define the terms “proximal” and “distal” here for a specific purpose but recognize their use relates to and is informed by wider critiques when discussing causality in epidemiology and public health [4, 5]. Climate change is only one amongst many huge societal challenges emerging from global environmental change. Addressing all such challenges requires the identification of actions which simultaneously protect ecosystems and human health and wellbeing in ways which are socially inclusive, sustainable and equitable, globally and across multiple generations. We recognize the important contribution of others (for instance the Millennium Ecosystem Assessment - MEA etc.) in identifying and exploiting ecosystem services as a bridge between the environmental science and public health communities and especially the issue of climate change and public health [6]. Developing this theme, we argue that pathways which appear distal to national public health concerns must be made explicit within national policy and decision making. In Scotland, holistic issue framing approaches were used to facilitate a richer interpretation of the environmental contribution in health and wellbeing and especially equity [7, 8]. This gave public health both traction and influence beyond its traditional territory resulting, e.g. in public health involvement in the creation of a place standard [9].

We argue that similar approaches can provide greater traction for public health in addressing local, national and international climate change and its determinants.

## Proximal and distal pathways to climate-related health effects

Global environmental change, including climate change, first engaged public health interest in the late twentieth century (e.g. [10–13]). In the UK, for example, the public health discourse on climate change, was conducted, initially at least, with a clear focus on environmental change taking place, or imminently anticipated, in that country. From the outset, concern centered on what the greater incidence and severity of flooding or more extreme weather in the UK would mean for the health of UK citizens [14]. In this paper, we propose the term *proximal* pathway to describe the process where a population’s health is imminently threatened or undermined through climate-related environmental change within its locality or within the borders of its own country and in ways readily comprehensible to that population (including its policy makers). Expressed in another way, from a national perspective, the *proximal* pathway is about the “here and now”. We recognize that the health effects arising in any country from the *proximal* pathway closely align with the direct health effects described by McMichael et al. in 1996 [15] and explored further by Butler and colleagues when first introducing four classifications of adverse health effects from climate change [16]. These broad classifications were reflected in later work [17, 18]. The International Panel on Climate Change (IPCC) [1, 19] has predominantly highlighted such direct impacts. The 2015 Lancet Commission [20] emphasizes the complexity of relationships between climate-related changes and health [19], distinguishing between direct and indirect impacts.

Here, we propose the terms *proximal* and *distal* pathways to better capture the true landscape of risks for those living in a particular location or country. The near term and lived proximal experience of climate change is related to encountering local and current changes in daily and seasonal weather patterns and extreme events. These manifestations, and their implications for health and wellbeing, can be widely understood and addressed (in part) by local responses, adding a sense of urgency and purpose to local adaptation and mitigation efforts.

Again, recognizing significant conceptual overlap with the “tertiary” health impacts described by others [16, 17], here we use the term distal pathway to describe three indirect routes by which climate change can affect human health, wellbeing and ecosystems. Such pathways are often mediated by both natural systems (e.g. disease vectors, water-borne diseases, air pollution) and human systems (e.g. occupational impacts, under-nutrition, and mental stress) [1].

Pathways to health and wellbeing may appear distal to a population in a particular location such as a country, for a combination of three reasons: they are considered temporally or spatially distal or the pathways themselves are particularly complex.

Many pathways are *temporally distal* because the extent of their effects on health and wellbeing will be experienced over time, or perhaps delayed for decades. The environmental changes which are component parts of these pathways are difficult to discern especially in average values of, for example, regional temperature change; rainfall intensity and aggregates; reduced snow and ice coverage; increased ocean acidity; and rising sea levels. All have the potential to affect health and wellbeing, often adversely, to a degree which depends not only on the future emission occurrence trajectory, but also on the success of local and global adaptive responses. Uncertainty, compounded by a limited understanding of how these (often incremental) changes can cause damage, means that policy makers and the public are often much more concerned about flooding, storms and heatwaves than about profound, widespread climatic changes. Again, using the example of the UK, climate-related sea level rise will eventually affect health in the UK [21, 22], but for the UK population, sea level rise is currently an example of a temporally distal pathway. Although many citizens in the UK can conceive some of what sea level rise might mean for their immediate lives, their economy and their health, the full societal impacts seem far down the line and remote [23, 24].

In contrast, for the people of the Maldives, sea level rise represents an acute (temporally) proximal pathway to an imminent risk [25].

Pathways from climate change to health and wellbeing can also be *spatially distal.* For any country and its population, these distal pathways relate to those environmental impacts which are happening or predicted to happen elsewhere. These can involve quite dramatic environmental changes in countries and regions beyond their borders, while little or no perceptible change in their own environment is experienced. Spatially distal pathways arise, for example, when areas elsewhere are damaged by extreme weather events leading to flooding and drought, or from more long term environment degradation and conflicts over scarce resources that result in displacement or permanent migration, or through the impact of distant events on the functioning of the global food system and therefore economic and physical access to food and local food security (see sections below on Food Security and Migration).

Finally, pathways are often distal because they are complex. Whether the climate-related environmental changes occur in one locality or concurrently across many regions, the pathways which lead to the negative impacts on health and wellbeing usually involve a complicated interplay of societal, economic and physical factors. This interplay can modify and often amplify risks and uncertainty.

The issue of climate change and pharmaceutical use offers an example of a climate-related health issue which is distal largely because it emerges from multiple and complicated interactions between social and environmental systems. Pharmaceutical use worldwide is likely to increase, and patterns of use change in response to climate-related rises in the burden of disease and the emergence of conditions unfamiliar in countries like the UK. These climate factors in combination with a global ageing demographic where there is a greater incidence of non-communicable and chronic disease will almost certainly mean greater use of commonly prescribed medicines, but also of other seldom used medicines [26]. The intentional or unintentional release of pharmaceuticals to the environment from human and veterinary use can be expected to impact on the structure and function of global and local ecosystems, undermining ecosystem services and, by extension, human health and wellbeing in many countries.

In the language of the IPCC Fifth Assessment Report, many of what we describe as distal pathways are “emergent from indirect, trans-boundary and long distance impacts of climate change” [27]. The long-term resource implications in responding to climate-related environmental change are rendered distal because they are also mired in complexity. For example, there is the current decision as to whether to allow fracking in the UK which will provide short term increases in fossil fuel access which in turn will increase global CO_2_ levels and local methane emissions, as well as causing significant local social, health and ecosystem impacts [28]. Furthermore, current resource decisions will have major impacts on their equitable national and international distribution and access in the future as climate change plays out over coming decades.

## A framework for distal and proximal health consequences of climate change

Unless communicated in more comprehensible and accessible ways, the distal pathways from climate change to health and wellbeing will certainly remain fractured and illogical to a significant and influential constituency, including policy makers and politicians. Yet it is often about more than communication. For policy and other decision makers, pathways that are distal in space or time are easier to disregard. The consequence is that key issues will be under-accounted for in decision-making. This has led to a growing demand to modernize public health around ecological principles. Sometimes termed “ecological public health”, the approach accords with the new importance attributed to these distal issues [3, 29, 30].

Yet it remains a major challenge to achieve recognition amongst the public and policymakers that the choices they make locally drive current and future climate-related environmental change wherever it occurs. Individual and societal choice forges the first links in every chain of events from human activities as drivers of climate change to immediate and distant health and wellbeing outcomes. However, if the necessary importance and priority are to be accorded to addressing climate change (and indeed all global environmental issues), a much broader constituency will need to have a much clearer understanding of the fundamental human reliance on natural ecosystems than currently appears to be the case. Such an understanding is central to making less opaque, particularly, the distal pathways from climate-related environmental change to health and wellbeing.

The use of simple conceptual models to think about and communicate human social complexity is well established in public health [31, 32]. In earlier work Morris et al., and Reis et al. [33, 34] have advocated the use of conceptual models to frame complex issues in the field of environmental health in a policy-relevant way. Morris et al. [33] modified the established **D**rivers **P**ressures **S**tate, **E**xposure, **E**ffect, **A**ction or “**DPSEEA**” model [35, 36] to better reflect social complexity in environmental health policy in Scotland [37, 38]. In part, this was achieved by capturing, within the model, the fact that a range of contextual factors can critically influence whether individuals are exposed to an aspect of environment and whether this exposure impacts on their health and wellbeing. Context is both an exposure and an effect modifier.

More recently, Reis et al. [34] developed an ecosystems enriched (**eDPSEEA**) model to make explicit how environmental health encompasses both the proximal environmental determinants of health and wellbeing, and also the impacts caused by anthropogenic damage to ecosystems. The **eDPSEEA** model incorporates the insights of the MEA [6] by explicitly linking ecosystem services (the benefits which humans derive from ecosystems) to human health and wellbeing within a notional chain of causation. It presents the health of humans and of ecosystems as intimately interconnected, and thus equally important to consider both as important outcomes.

The MEA [45] achieved a more inclusive and policy-relevant representation of the wider importance of ecosystem services by identifying four different types of ecosystem services: provisioning, regulating, cultural and supporting. The MEA also projected how ecosystem services impact on human wellbeing, whether through the supply of material goods or through supporting social relations, security and freedom of choice and undermining health itself. Since then, the concept and structure of ecosystem services and their relationships with, and relevance for, humanity have been widely discussed. Fisher et al. [37] distinguish between *intermediate* and *final* ecosystem services, while De Groot et al. [38] relate ecosystem functions and the services they provide in a comprehensive, integrated framework, incorporating earlier work by Daily et al. [39, 40]. A common feature of many ecosystem services (ES) definitions, e.g. as used in the MEA and the UK National Ecosystem Assessment [41] is that some services provide direct benefits (provisioning, regulating and cultural ES). In contrast others, like nutrient recycling or soil formation underpin ecosystem function (supporting ES).

**Fig. 1:**
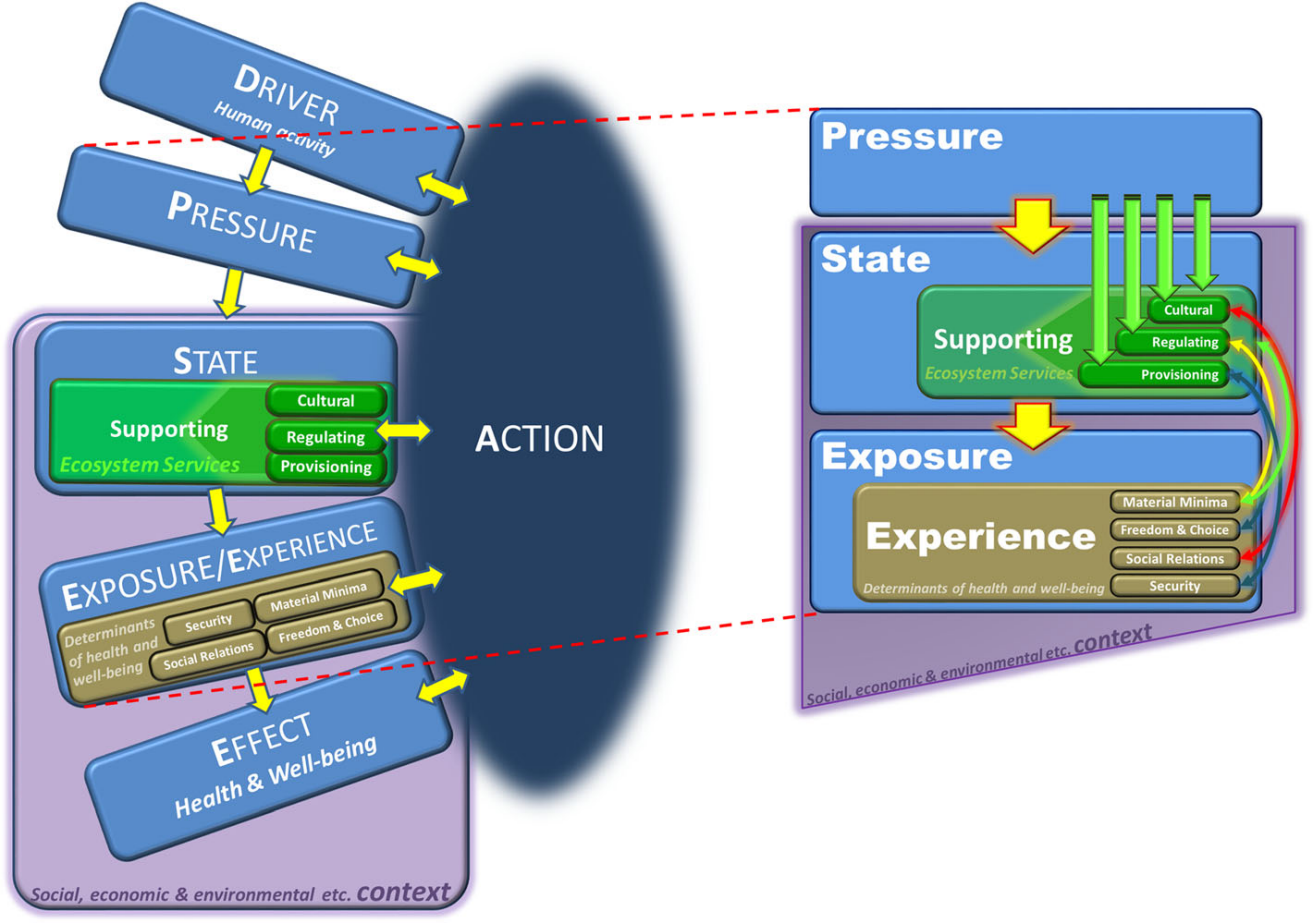
The Ecosystems Enriched **eDPSEEA** Model [34]

Figure 1 embeds the concept of ecosystem services and their relationships with both human health and the proximal and distal determinants of human health and wellbeing more broadly.

It has been observed that “all models are wrong but some are useful” [42]. Yet, In addition to promoting mechanistic understanding, the process and the product of populating simple conceptual models, such as eDPSEEA and others, can clarify both the distal and proximal pathways through which climate change can affect health and serve as a tool for engagement with stakeholders [34].

## Proximal and distal stressors: Food systems and mobility

It is evident that both short term proximal climate-related stressors, and the more remote, longer term, indirect distal stressors are acting together to generate threats to public health and wellbeing in all locations, particularly with deprived populations globally. There is a growing number of examples of health inequality issues as climate change increasingly affects global food security and population migration.

Individuals and socio-economic groups in local environments are affected by a combination of proximal and distal effects, some immediate, but others subsequently translated by economic, biogeochemical and resource flow mechanisms. These mechanisms have been elaborated by Adger et al. [43] as teleconnections linking vulnerabilities across space and time, and by Liu et al. [44, 45] as connections between sending, receiving and spillover systems. Here, we develop insights using a dichotomy between the distal and the proximal pathways from environmental change to human health and wellbeing, recognizing the inherent complexity of most interactions. Macro and micro level processes continually interact and are tele-connected through systemic environmental processes, through the flows of material and mobility of populations around the world, and, importantly, through market and economic linkages [43].

### Climate change and distal food security

Food, nutrition and agricultural trade are potentially sensitive to climatic changes [1, 46]. Rising levels of CO_2_ both lead to a changing climate and can reduce the nutritional quality of crop production [47]. Relative to an unchanging climate, yields of principal agricultural crops are already being affected globally, and have the potential to decline without major adaptations in technology and water use efficiency [1, 46, 48]. These changes are spatially sensitive, with risks of yield decrease likely to be greater in the hotter parts of the world [49]. However, there is considerable scope for the food and trade-system to adapt to climate change [48]. This might be achieved via changes in the area of production (expansion of which may exacerbate climate change by liberating carbon in land conversion), and through impacts on trade and prices. However, given the complexity of the system, it is not clear how the multiple potential drivers of food availability and price will interact around issues such as: food for feed [50]; biofuels [51]; carbon pricing [52]; water availability [53]; competition for land and other resources [54]; and the need for agriculture to be sustainable [55]. Increasing weather variability may lead to short term unexpected shocks to supply [1, 46] that create significant volatility in food prices, impacting on the wellbeing of food insecure populations in all parts of the world.

Variation in prices driven by weather-related impacts have accentuated price shocks and created localized food shortages [56]; both factors impact most, the poor sections of populations. For example, in the UK, analysis of purchases following the 2007/8 global commodity food price spike show that as prices increased, households purchased 4.2% less food [57] and bought lower quality alternatives. The greatest impact was on the poorest income decile: they spent 17% more in 2011 compared to 2007, so their relative food bill increased by 40% more than the UK average. On a global basis, food price spikes, driven by weather in the main bread-basket regions, directly impact market prices in the import-dependent low income countries, as well as indirectly influencing the food aid donated by the rich world. As a result, in sub-Saharan Africa particularly, the number of hungry increased following the 2007/8 and 2010/11 food price spikes. Furthermore, the food price spike of 2010/11 has been estimated to have pushed >40 million people globally below basic needs poverty line in those years [58]. Thus, weather impacts from climate change are likely to impact nutritional and future health status in all parts of the world and among consumers as well as producers [55].

While food prices provide a proximal link between food security and climate change, the distal implications of climate change are profound. The growth of demand for food is driven by rising population size and wealth, and the need for sustainability. In many analyses demand is regarded as exogenous, driven by relationships with increasing wealth [59], to which interventions need to be directed. However, the relationship between food and health are likely to shape trends in demand [50, 59], and thus affect global agricultural production.

### Climate change proximal and distal implications through migration and mobility

Climate changes involve spatial changes to economic and environmental systems that will prompt proximal and distal demographic responses. Fundamentally climate change will have an impact on where people live and on the decisions they make about moving from one location to another. Migration is a central element of economic and demographic change everywhere in the world. In effect, migration flows at the aggregate level are driven principally by differences in economic activity across space and time, though all individual decisions involve social, cultural and demographic dimensions. Some elements of the relative attractiveness of different areas, and hence the demand for migration, are sensitive to weather and climate. Hence resource scarcity, the availability of ecosystem services, and issues of security and hazard, all factor in the relative attractiveness of places and decisions to move between them [60–63].

Climate changes have proximal and distal impacts on different types of migration. Displacement of populations from their place of residence as a result of extreme events is most often temporary and undertaken involuntarily, but has major public health and policy consequences. In the UK, for example, flood events temporarily displace people from their homes, often for months after events [64]. The impacts of Hurricanes Katrina and Rita in Louisiana and New Orleans in 2005 showed that displacement of populations from the flood impacts lead to very divergent patterns of who returned and who permanently migrated: wealthier populations predominantly returned while poorer populations more frequently moved away permanently, thus changing the demographics of the whole region in the long term [65].

Climate change-induced resource scarcity reduces the potential for capital accumulation in resource-sensitive economies, and thus has a potential negative impact on the mobility potential of sections of the population who do not have the resources necessary for migration. Hence, populations may experience a poverty-immobility nexus, where increased mobility would be necessary for effective adaptation. In addition, migration trends of populations moving into expanding cities throughout developing and emerging economies means that a growing number of populations are more exposed to weather and climate hazards in those migration destination areas.

A further interaction between migration and climate change is forced migration due to conflict. This type of migration is also involuntary, and has implications in both conflict areas and population-receiving areas. The direct links between climate risks and conflict risks are not well established, yet the issue of attribution and causation is not the most relevant issue [66, 67]. The IPCC Fifth Assessment Report concludes that climate change impacts are likely to exacerbate poverty in resource-sensitive regions and that since poverty is a principal driver and predicator of violent conflict, the risks of climate change amplifying conflict risks in future are real [66]. Conflict itself has significantly differential effects on the ability of populations to relocate from conflict zones [68, 69]. Climate change, if it is to affect conflict risk, does so through expanding poverty as a principal cause of insecurity and conflict. Hence, in theory, there is a plausible route for increased risk in conflict-prone areas of the world over the incoming decades, in the absence of efforts for development and relief of the underlying causes of conflict in those regions [66, 67].

The principal form of migration globally, however, continues to be the movement of populations to urban centers within their national borders. In terms of absolute numbers, this trend is apparent and stark in Asia and Africa in particular [70, 71]. Geographically, these migration trends are fueling trends of population movement towards coasts, and movement away from dry land and mountain environments [72]. This dominant migration trend, in terms of numbers, creates significant environmental and public health challenges. The migrant populations moving into cities are differentially exposed to climate hazards in those places: low income migrant communities are often located in flood prone zones or areas susceptible to landslides. Migrant populations cluster in areas with low air quality or in slums with lack of access to sanitation or clean water [60, 62]. Hence migration trends exacerbate environmental health risks: as many people are moving towards risks as moving away from them. These processes have both distal and proximal dimensions.

## Conclusions

A weight of evidence suggests that climate-related environmental change in one part of the world will have systemic health and wellbeing impacts elsewhere at some point. Complex global interconnectivities underpin the pathways which are spatially and temporally distal. Vulnerability to health effects in geographically distant places is translated to individuals and communities by economic, social, ecological, biogeochemical, and resource flow mechanisms.

Future policies and interventions to deal with these risks need to account for how those risks are spatially and socially differentiated, and how their accessibility is dependent on a range of social and cultural contexts, such that the benefits of those interventions are widespread [2, 20]. Similarly, the mitigation of climate change through decarburization of energy and altered economic systems have the potential to bring about significant benefits to health and wellbeing, especially if these are widely distributed. Despite sentinel attempts over time by various commentators [see for example [15–18], there is still a need to help people in specific locations or countries (including policymakers) to understand and communicate climate-related health threats on vastly expanded temporal and spatial scales.

The concept of ecosystem services and recent representations of their links to human health and wellbeing [6, 34] demonstrate important relationships in many chains of causation. Both the benefits and dis-benefits of globalization are unevenly distributed between and within countries and regions, and are invariably socially patterned and stratified to impact the most deprived. The complexity of proximal and distal pathways, suggests the need for a set of rapidly evolving novel qualitative and quantitative evidence and analysis techniques associated with the growth of big data in environment and human health research [73] The linking of ecosystem services to human health and wellbeing can be an important component in operationalizing a new truly ecological public health. Communicating to a wide and diverse audience, that fostering better human health and wellbeing depends upon, and is intimately linked to, the changing state and sustainability of the Earth’s geochemical and ecological systems, remains one of the greatest challenges of our time.

## Additional file

Additional file 1: Open peer review. (PDF 469 kb)
