# Supplementary material for: Scoping the proximal and distal dimensions of climate change on health and wellbeing
Source: Environ Health. 2017 Dec 5;16(Suppl 1):116. doi: 10.1186/s12940-017-0329-y (PMC5773875; doi:10.1186/s12940-017-0329-y)
Supplement: Additional file 1: — Open peer review. (PDF 469 kb) [file 12940_2017_329_MOESM1_ESM.pdf]

Reviewer reports

Title: Scoping the Proximal and Distal Dimensions of Climate Chang on Health and Wellbeing

Reviewer 1: Hilary Graham

This is an interesting and clearly-written paper addressing what many regard as the major 21st century threat to public health. It does not present new findings and perspectives; its primary concern, instead, is to bring together existing evidence and frameworks in ways that highlight the multiple pathways through which the health effects of climate change are mediated. As I read the Abstract, this task is undertaken in order to aid public health planning and the conceptualization of interventions to mitigate and adapt to changing environmental conditions.

There are three areas where the paper could be strengthened.

Firstly, I would recommend that a clearer statement of aims and methods is given in the Abstract and then in the body of the paper. Currently, the Abstract tells the reader what the paper does ('reviews evidence' 'presents a conceptual model') but does not explain why and to what end these exercises are being undertaken. A statement of aims would help here, and should also be included in the Introduction section of the paper. Relatedly, the Abstract provides no information on methods (how the evidence was accessed and assessed etc.) – and again the body of the paper has no discussion of the methods used to scope and review the evidence and scope and review potentially-relevant conceptual models. Food systems and migration feature as case studies in the later part of the paper; signally these foci in the Abstract and explaining them in the Introduction would again be helpful.

Related to this first point, it is not altogether clear where the originality of the paper lies. A crisper Abstract and Introduction would ensure that the reader is left in no doubt about this. To explain: with respect to the evidence review part of the paper, there are many existing reviews and reports summarizing evidence on health impacts of climate change and the complex pathways through which these impacts occur, including both those cited in the paper and others. So the reader may be asking 'what does the paper add?' With respect to the conceptual model, the one presented is taken from the authors' previously-published work – again the reader may therefore ask 'where is the originality?'

Secondly, the concepts of 'proximal' and 'distal' anchor the paper. These are very familiar terms within social epidemiology and public health – and have been the subject of influential critiques. The authors should provide definitions of what they mean by the terms, particularly as they use them to refer to various points along the causal chain: proximal and distal determinants (and stressors and threats), proximal and distal pathways, proximal and distal experiences and proximal and distal effects. If they wish to discuss how they usage of the terms relates to and/or is informed by wider critiques of the terms,

examples include Tony McMichael's 1999 paper 'Prisoners of the Proximate' and Nancy Krieger's 2008 paper 'Proximal, Distal, and the Politics of Causation'.

Thirdly, there is relatively little attention given to what the evidence review and conceptual model mean in practice for public health planning and interventions (this was signaled as part of the paper's remit in the Abstract). I was expecting a section discussing how a focus on the 'proximal' and 'distal' domains helped with policy development and evaluation, particularly given the authors' important emphasis in the Introduction on equity and sustainability across societies and generations. I would suggest either this aspect of the paper is strengthened, or the Abstract and Introduction are revised to downplay it.

In addition to these three areas, I would encourage the authors to address two other points. First, the paper introduces an ecosystem focus (2nd para of the Introduction). Does the term need defining and this focus explaining – and then drawn more clearly through the rest of the paper? Secondly, while references are cited to support many statements in the paper, there are a number of fairly bold statements that have no evidence cited in their support.

Declaration of competing interests: No competing interests to declare.

Reviewer 2: Colin Butler

Thanks for the chance to comment on this paper, "Scoping the Proximal and Distal Dimensions of Climate Change on Health and Wellbeing".

There was much within it with which I generally agree, but the antecedents of many of the concepts here should be better acknowledged.

There is substantial conceptual overlap, with earlier work, in particular see:

Butler C.D., Corvalán, C.F. and Koren, H.S. (2005): Human health and well-being in global ecological scenarios. *Ecosystems* **8**(2): 153-64.

Butler C.D., Harley D. (2010) Primary, secondary and tertiary effects of eco-climatic change: the medical response. *Postgraduate Medical Journal* 86:230-234.

Butler C.D. (2014) Climate Change and Global Health: a new conceptual framework CAB Reviews: *Perspectives in Agriculture, Veterinary Science, Nutrition and Natural Resources* 9(27) doi: 10.1079/PAVSNNR20149027

I know I am the first author of these three (all peer reviewed) and it perhaps seems brash to highlight my own work, but there is much overlap and I really think the lead author should read all three and cite at least one. There is also an edited book, building on the conceptual framework (Butler C.D., (2016), Climate Change and Global Health. CABI, Wallingford, UK, 318+xxiv pages (softcover edition)). Approximately 1/5th of this book discusses content very similar to what you have called "distal" – I called it "tertiary".

Otherwise, the readers of the final piece may not recognise this overlap and thus overestimate its conceptual originality. It is perhaps also worth mentioning that the final section of the health chapter in the recent IPCC report made a similar statement about what you call distal effects, though, unfortunately, without providing a citation.

I also think it would be worth stating that none of these conceptual models are complete, ideal or perfect .. rather, they are like different map projections (Mercator, Peters etc) with strengths and weaknesses. Ultimately the value of these mental models depends too on the mind of the reader; i.e. the existing concepts and thoughts with which readers of this paper build on.

More specific comments are in the attached annotated file (**below**). Colin Butler July 10, 2016

Declaration of competing interests:

- Have you in the past five years received reimbursements, fees, funding, or salary from an organisation that may in any way gain or lose financially from the publication of this paper, either now or in the future?

Yes, I published an edited book using the "primary, secondary and tertiary" health framework, from which I derive royalties. This framework is similar to that in the reviewed paper

- Do you hold any stocks or shares in an organisation that may in any way gain or lose financially from the publication of this paper, either now or in the future?

I hold stocks, personally and in my retirement fund, whose value should rise if climate change is taken more seriously

- Do you have any non-financial competing interests in relation to this paper?

Only that I am anxious about the long term effects of climate change

Scoping the Proximal and Distal Dimensions of Climate Change on Health and Wellbeing

Morris GP[a], Reis S[b,a], Beck SA[c], Fleming LE[a], Adger WN[d], Benton TG[e], Depledge MH[a]

Author Affiliations

[a]European Centre for Environment and Human Health, University of Exeter Medical School, Truro, Cornwall, UK

[b]NERC Centre for Ecology & Hydrology, Bush Estate, Penicuik, Midlothian UK

[c]NHS Health Scotland, Meridian Court, Cadogan Street, Glasgow, UK

[d]Geography, College of Life and Environmental Sciences, University of Exeter, Rennes Drive, EX4 4RJ, Exeter, UK

[e]UK's Global Food Security Programme and School of Biology, University of Leeds, UK

Corresponding Author

Professor George P Morris

European Centre for Environment and Human Health

University of Exeter Medical School C/o Knowledge Spa RCHT

Truro, Cornwall, TR1 3HD

UK

Email: geomorris55@hotmail.co.uk

Abstract

The impacts of climate on health and wellbeing occur in time and space and through a range of indirect, complicated mechanisms. This diversity of pathways has major implications for national public health planning and influence on ~~the conceptualisation of~~ interventions that might help to mitigate and adapt to rapidly changing environmental conditions, ~~s.~~ nationally and internationally. ~~-~~This paper ~~reviews~~ draws upon evidence from public health and adverse impact studies across climate science, hydrology, agriculture, public health, and the social sciences. It presents a conceptual model to support decision-making by recognizing ~~of~~ -both the proximal and distal pathways from climate-induced environmental change to national health and wellbeing. ~~effects of climate change, relating ecosystem services to health and wellbeing outcomes.~~ The proximal and distal ~~elements of~~ pathways associated with food security, and migration and mobility illustrate the diverse climate change influences in different geographic locations over different timescales. We argue that greater realization and articulation of proximal and distal ~~relationships~~ pathways should radically alter how climate change is addressed as a national and international public health challenge.

Comment [s1]: I like this proposed scope

Comment [GM2]: We thank the reviewer for this supportive comment.

Comment [CB3]: agree

Key Words

ecosystems, food, nutrition, mobility, migration, concepts, stakeholder engagement, theoretical frameworks, ecosystem services.

Acknowledgements

Publication of this article was funded by the UK Living with Environmental Change Network http://www.lwec.org.uk/about. Contributory research was funded in part by the National Institute for Health Research Health Protection Research Unit (NIHR HPRU) in Environmental Change and Health at the London School of Hygiene and Tropical Medicine in partnership with Public Health England (PHE), and in collaboration with the University of Exeter, University College London, and the Met Office; the UK Medical Research Council (MRC) and UK Natural Environment Research Council (NERC) for the MEDMI Project; and the European Regional Development Fund Programme and European Social Fund Convergence Programme for Cornwall and the Isles of Scilly (University of Exeter Medical School)

None of the authors has any competing interests in the manuscript.

Introduction

The effects of global climate change are now observable in every part of the world. Scientific assessments suggest that nowhere will be immune to the future threats climate change poses to human health and wellbeing [1]. Remarkably, many of the indirect adverse health impacts driven by climate-related ecological disruption and their consequences remain to be explored. Crop failures and shifting patterns in disease vectors are remote from current decision-making on energy systems and the aggregating emissions of greenhouse gases. Impacts emerge both from the physical and ecological changes across the globe, and from the societal responses such as geographic and social displacement of populations in conditions of prolonged drought or of severe and persistent flooding. Behaviours and lifestyles, as well as health, social and economic inequalities, will be profoundly affected by climate change [2,3].

Comment [CB4]: not US spelling

~~We~~ This paper does not seek to systematically review the health impacts of climate change but rather to reinforce the need for any country or community to better capture and communicate its true public health implications in a policy-relevant way. We focus here on the adverse impacts ~~of climate change~~ of climate change on health and wellbeing, through what we define as proximal and distal pathways. We adopt and define the terms “proximal” and “distal” here for a specific purpose but recognize their use relates to and is informed by wider critiques when discussing causality in epidemiology and public health [4,5]. Climate change is only one ~~yet climate change is but one~~ amongst many huge societal challenges emerging from ~~the complex interconnected effects of~~ global environmental change. Addressing all

such challenges requires the identification of actions which simultaneously protect ecosystems and human health and wellbeing in ways which are socially inclusive, sustainable and equitable, globally and across multiple generations. We recognize the important contribution of others (MEA etc) in identifying and exploiting ecosystem services as a bridge between the environmental science and public health communities and especially the issue of climate change and public health [~~24~~6]. Developing this theme, we argue that pathways which appear distal to national public health concerns must be made explicit within national policy and decision making. In Scotland, holistic issue framing approaches were used to facilitate a richer interpretation of the environmental contribution in health and wellbeing and especially equity [~~22, 23~~7, 8]. This gave public health traction and influence beyond its traditional territory resulting, e.g. in public health involvement in the creation of a place standard [9].

We argue that similar approaches can provide greater traction for public health in addressing local, national and international climate change and its determinants.

> **Comment [CB5]:** this appears to be first use of this acronym, but without definition

Proximal and distal ~~stressors~~ pathways to climate-related health effects

> **Comment [GM6]:** We have clarified this heading in the context of the subsequent changes in the section, as we reserve the terms, proximal and distal for describing pathways. The Proximal is about the "Here and Now" the Distal is about the "then and there"!

Global environmental change, including climate change, first engaged public health interest in the late 20th century (e.g. [~~4-7~~10-13]). In the UK, for example, the public health discourse on climate change ~~centered, initially at least~~, was conducted, initially at least, with a clear focus on environmental change taking place, or imminently anticipated, in that country. From the outset, concern centered~~,~~ on what

the greater incidence and severity of flooding or more extreme weather in the UK would mean for the health of UK citizens [819]. In this paper we propose the term "proximal" pathway to describe the process where a population's health is imminently threatened or undermined through climate-related environmental change within its locality or within the borders of its own country and in ways readily comprehensible to that population (including its policy makers). Expressed in another way, from a national perspective, the proximal pathway is about the "here and now". on the local and immediate implications of flooding and extreme weather [8], We recognize that the health effects arising in any country from the proximal pathway closely align with the "direct" health effects described by McMichael et al. in 1996 [14] and explored further by Butler and colleagues when first introducing four classifications of adverse health effects from climate change [15]. These broad classifications were reflected in later work [16, 17]. focusing on proximal, direct and often localised health threats from climate (i.e. impacts which are near in time and space). The International Panel on Climate Change (IPCC) [1,198] has predominantely highlighted disuch direct impacts. The 2015 Lancet Commission [1020] emphasises the complexity of relationships between climate-related changes and health [19], distinguishing between direct and indirect impacts.

Here, we propose the terms proximal and distal pathways to better capture/approach accurately describe the true landscape of risks for those living in a particular location or country. The near term and lived proximal experience of climate change is related to encountering local and current changes in daily and seasonal weather patterns and extreme events. These manifestations, and their implications for health and

Comment [CB7]: Just a suggestion

Comment [s8]: Here, direct is introduced –is this a synonym for "proximal"?

Comment [GM9]: We have revised this sentence to be more clear, see as well revisions of the heading and rest of this section

Comment [s10]: agree

Comment [s11]: this claim is an over reach

Comment [GM12]: Agreed. We have rephrased and qualified our statement in the context of the revised text.

Comment [s13]: this seems like "direct" in the classification of McMichael AJ, Haines A, Slooff R, Kovats S (Eds): *Climate change and human health*. World Health Organization, Geneva (1996). and also Butler et al 2005, 2010, and Butler 2014 (etc)

Comment [GM14]: Agreed there is overlap but they're not the same. We have added text and revised to better reflect this.

wellbeing, can be widely understood and addressed (in part) by local responses, adding a sense of urgency and purpose to local adaptation and mitigation efforts.

Again, recognizing significant conceptual overlap with the "tertiary" health impacts described by others [15, 16], here we use ~~We use~~ the term distal pathway to ~~to~~ describe three indirect routes ~~pathways~~ by which climate change can affect ~~both~~ human health, ~~and~~ wellbeing and ecosystems. Such pathways are often mediated by both natural systems (e.g. disease vectors, water-borne diseases, air pollution) and human systems (e.g. occupational impacts, under-nutrition, and mental stress) [1].

Comment [CB15]: Again, you may want to consider inverted commas.. alas, the term is not well-known, and not necessarily self-explanatory. However, up to you.

Pathways to health and wellbeing ~~are usually distal~~ may appear distal to a population in a particular location such as a country, for a combination of three reasons: they are considered temporally or spatially distal or the pathways themselves are particularly complex.

Comment [s16]: yet heatwaves and disasters are usually proximal

Comment [GM17]: Agreed, but we argue that they can be distal too because in our terms, proximal and distal are relative to the location in question. See revised text which addresses this more clearly.

Many pathways are *temporally distal* because the extent of their effects on health and wellbeing will be experienced over time, or perhaps delayed for decades. The environmental changes which are component parts of these pathways ~~lead to such impacts~~ are difficult to discern especially in average values of, for example, regional temperature change; rainfall intensity and aggregates; reduced snow and ice coverage; increased ocean acidity; and rising sea levels. All have the potential to affect health and wellbeing, often adversely, to a degree which depends not only on the future emission occurrence trajectory, but also on the success of local and global adaptive responses. Uncertainty, compounded by a limited understanding of how these (often incremental) changes can cause damage, means that policy makers

and the public are often much more concerned ~~over~~ about flooding, storms and heatwaves than about profound, widespread climatic changes. Again, using the example of the UK, climate-related s~~S~~ea level rise will eventually affect health in the UK [21, 22], but for the UK population, sea level rise is currently an example of a temporally distal pathway. Although many citizens in the UK can conceive some of what sea level rise might mean for their ~~society~~immediate lives, their economy and their health, ~~it~~the full societal impacts ~~still~~ seem~~s~~ far down the line and remote [23, 24].

In contrast, for the people of the Maldives, sea level rise represents an acute (temporally) proximal pathway to an imminent risk. [25]. ~~is a very obvious example of a temporally distal pathway. Most can conceive some of what it might mean for society, the economy and health, but it still seems far down the line and remote.~~

Pathways from climate change to health and wellbeing can also be *spatially distal.* For any country and its population, these distal pathways relate to those environmental impacts which are happening or predicted to happen elsewhere. These can involve quite dramatic environmental changes in countries and regions beyond their borders, while little or no perceptible change in their own environment is experienced. Spatially distal pathways arise, for example, when areas elsewhere are damaged by extreme weather events leading to flooding and drought, or from more long term environment degradation and conflicts over scarce resources that result in displacement or permanent migration, or through the impact of distant events on the functioning of the global food system and therefore economic and physical access to food and local food security (see sections below on Food Security and Migration)~~.~~

Comment [GM18]: We have added further references to underpin this point.

Comment [s19]: Not so remote to some populations

Comment [GM20]: We have revised the text to clarify this.

Comment [s21]: perhaps such as in Syria at the moment, compared to (say) the UK

Comment [GM22]: I am sure there is a climatic dimension to the Syria thing, the need to explain it to the reader would introduce extra words which we can't afford and it would go beyond the scope and focus of this paper to fully discuss this complex matter.

Comment [CB23]: OK

Comment [s24]: take a super-typhoon in The Philippines: spatially distal to the UK, direct and immediate in The Philippines. This is surely obvious and I don't know why you are saying it

Comment [GM25]: I accept the observation that it is obvious and may not need to be said but I thought it might help emphasis that one community's distal pathway is another's proximal pathway.

Finally, pathways are often distal because they are complex. Whether the climate-related environmental changes occur~~s~~ in one locality or concurrently across many regions, the pathways which lead to the negative impacts on health and wellbeing usually involve a complicated ~~n~~ ~~unfamiliar~~ interplay of societal, economic and physical factors. This interplay can modify and often amplify risks and uncertainty.

The issue of climate change and pharmaceutical use offers an example of a climate-related health issue which is distal largely because it emerges from multiple and complicated interactions between social and environmental systems. Pharmaceutical use worldwide is likely to increase, and patterns of use change in response to climate-related rises in the burden of disease and the emergence of conditions unfamiliar in countries like the UK. These climate factors in combination with a global ageing demographic where there is a greater incidence of non-communicable and chronic disease will almost certainly mean greater use of commonly prescribed medicines, but also of other seldom used medicines [~~11~~26]. The intentional or unintentional release of pharmaceuticals to the environment from human and veterinary use can be expected to impact on the structure and function of global and local ecosystems, undermining ecosystem services and, by extension, human health and wellbeing in many countries.

In the language of the IPCC Fifth Assessment Report, many of what we describe as distal pathways are "emergent from indirect, trans-boundary and long distance impacts of climate change" [~~12~~27]. The long term resource implications in responding to climate-related environmental change are rendered distal because they are also mired in complexity. For example, there is the current decision as to whether to allow fracking in the UK which will provide short term increases in fossil

Comment [s26]: unfamiliar to who? To PH audiences? If you mean PH then your comment on why this is unfamiliar would be useful

Comment [GM27]: I suppose public health does pride itself on its capacity to appreciate socio-ecological complexity so "unfamiliar" is not a useful inclusion here. I simply meant that the interplay is complicated and difficult for people to predict or "get their head round" . However, we have taken this out as it was not immediately clear to a reader.

Comment [GM28]:

Comment [CB29]: OK

Comment [s30]: true

Comment [s31]: because? State reason here (rather than below) – ageing, affluence, increased power of pharmaceuticals? OR – do you mean only from CC? But, if CC leads to more poverty why will it increase?

Comment [GM32]: We address this in the next section, which should be in sufficient proximity to the points addressed here.

Comment [CB33]: OK -unfamiliar with Ref 26 and I'm skeptical, but I'm not going to draw a line in the sand over this!

fuel access~~, but~~ which in turn will increase global CO_2 levels as well as causing significant local social, health and ecosystem impacts [~~13~~28]. Furthermore, current resource decisions will have major impacts on their equitable national and international distribution and access in the future as climate change plays out over coming decades.

A framework for distal and proximal health consequences of climate change

Unless communicated in more comprehensible and accessible ways, the distal pathways from climate change to health and wellbeing ~~seem set~~will certainly ~~to~~ remain fractured and illogical to a significant and influential constituency, including policy makers and politicians. Yet it is often about more than communication. For policy and other decision makers, pathways that are distal in space or time are easier to disregard. ~~Key~~ The consequence is that key issues will be under-accounted for in decision-making. Th~~e~~is has led to a ~~term~~ growing demand to modernize public health around ecological principles. Sometimes termed “ecological public health”, – the approach accords with the new importance attributed to these distal issues ~~“ecological public health” now describes a growing demand to modernise public health around ecological principles~~ [3,~~14,15~~29, 30].

How to achieve recognition amongst the public and policymakers that the choices they make drive current and future climate-related environmental change wherever it occurs is still a major challenge. Individual and societal choice forges the first links in every chain of events from human activities as drivers of climate change to immediate and distant health and wellbeing outcomes. However, if the necessary importance and priority are to be accorded to addressing climate change (and

Comment [CB34]: And CH4?

Comment [s35]: Agree, BUT, not only because of difficulty in communicating but also resistance of public and policy makers to consider effects that are distal in space and time

Comment [GM36]: Agreed. We have added/rephrased to address this comment.

Comment [CB37]: Sentence is a bit awkward

indeed all global environmental issues), a much broader constituency will need to have a much clearer understanding of the fundamental human reliance on natural ecosystems than currently appears to be the case. Such an understanding is central to making less opaque, particularly, the distal pathways from climate-related environmental change to health and wellbeing.

The use of simple conceptual models to think about and communicate human social complexity is well established in public health [~~16~~31,~~17~~32]. In earlier work Morris et al., ~~,~~and Reis et al. ~~and colleagues~~ [~~18~~33,~~19~~34] have advocated the use of conceptual models to frame complex issues in the field of environmental health in a policy-relevant way. Morris et al [~~18~~33] modified the established **D**rivers **P**ressures **S**tate, **E**xposure, **E**ffect, **A**ction or "**DPSEEA**" model [~~20~~35, ~~21~~36] to better reflect social complexity in environmental health policy in Scotland [~~22~~37, ~~23~~38]. In part, this was achieved by capturing, within the model, the fact that a range of contextual factors can critically influence whether individuals are exposed to an aspect of environment and whether this exposure impacts on their health and wellbeing. Context is both an exposure and an effect modifier.

More recently, Reis et al. [~~19~~34] developed an ecosystems enriched (**eDPSEEA**) model to make explicit how environmental health encompasses both the proximal environmental determinants of health and wellbeing, and also the impacts caused by anthropogenic damage to ecosystems. The **eDPSEEA** model incorporates the insights of the Millennium Ecosystems Assessment (MEA) [~~2~~46] by explicitly linking ecosystem services (the benefits which humans derive from ecosystems) to human health and wellbeing within a notional chain of causation. It presents the health of

> **Comment [CB38]:** Acronym should be defined earlier

both humans and of ecosystems as intimately interconnected, and thus equally important to consider both as important outcomes.

The 2005 MEA [2446] achieved a more inclusive and policy-relevant representation of the wider importance of ecosystem services by identifying four different types of ecosystem services: provisioning, regulating, cultural and supporting. The MEA also projected how ecosystem services impact on human wellbeing, whether through the supply of material goods or through supporting social relations, security and freedom of choice and undermining health itself.. Since then, the concept and structure of ecosystem services and their relationships with, and relevance for, humanity have been widely discussed. Fisher et al. [2537] distinguish between *intermediate* and *final* ecosystem services, while De Groot et al. [38] relate ecosystem functions and the services they provide in a comprehensive, integrated framework, incorporating earlier work by Daily et al. [39, 40]. highlighting A common feature of many ecosystem services definitions, e.g. as used in the MEA and the UK National Ecosystem Assessment [41] is that some services provide direct benefits (provisioning, regulating and cultural ES), whereas others underpin ecosystem function (supporting ES).

Comment [CB39]: Word missing? "it"?

Comment [s40]: The conceptual framework dates to 2003; you can cite a 2005 paper but better to not include the date here in the text

Comment [GM41]: I have read the document and of course, Colin is absolutely correct. The easy way out is, as he helpfully suggests, to reference it to the MEA as we have done but omit the date from the text.

Comment [s42]: In the MA health was considered one of the 5 main components of HWB

Comment [GM43]: Think I've covered this.

Comment [CB44]: Sentence not so elegant with "ecosystem services" and two mentions of "ES". But, if you can live with it..

Comment [s45]: One of the main ES services was "supporting", so I would prefer the text to be rephrased to a way which does not give undue credit to Fisher et al's originality; which from this description seems to be highlighting something long understood.

Comment [GM46]: I think the reviewer has a good point here. The paper which appears to introduvce the framework and the mechanisms linking ecosystems services to wellbeing which the MEA built on was one of Colin Butler's from 2003 where supporting services were highlighted....

Comment [RS47]: We have rephrased and provided additional references to strengthen this point.

Comment [CB48]: OK

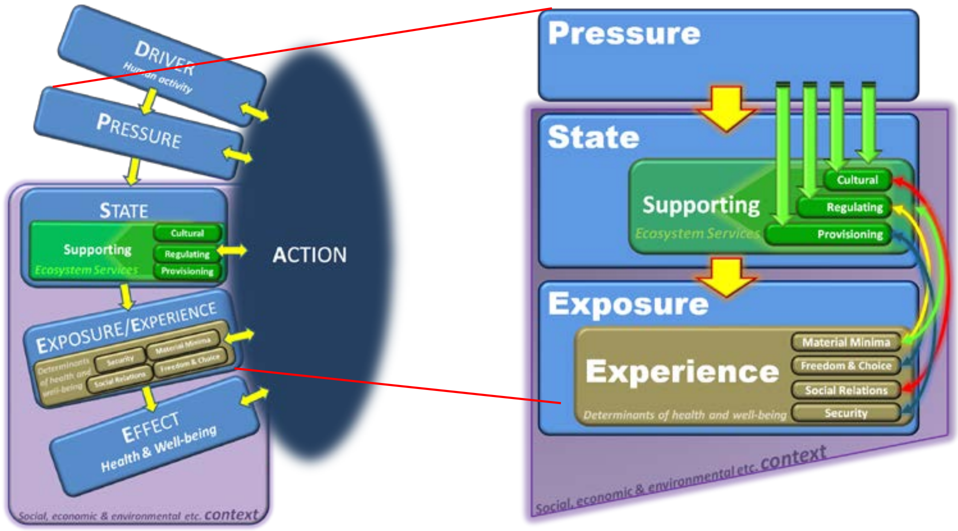

Figure 1: The Ecosystems Enriched **eDPSEEA** Model [1934]

Figure 1 embeds the concept of ecosystem services and their relationships with both human health and the proximal and distal determinants of human health and wellbeing more broadly.

It has been observed that "all models are wrong but some are useful" [43]] In addition to promoting mechanistic understanding, theThe process and the product of populating simple conceptual models, such as eDPSEEA and others, can clarify both the distal and proximal pathways through which climate change can affect health and serve as a tool for engagement with stakeholders [1934].

Proximal and distal stressors: food systems and mobility

It is evident that both short term proximal climate-related stressors, and the more remote, longer term, indirect distal stressors are acting together to generate threats to public health and wellbeing in any all locations, particularly with deprived populations globally. There is a growing number of examples of health inequality

issues as climate change increasingly affects global food security and population migration.

Individuals and socio-economic groups in local environments are affected by a combination of proximal and distal effects, some immediate, but others subsequently translated by economic, biogeochemical and resource flow mechanisms. These mechanisms have been elaborated by Adger *et al.* [~~26~~44] as teleconnections linking vulnerabilities across space and time, and by Liu et al. [~~27~~45, ~~28~~46] as connections between sending, receiving and spillover systems. Here, we develop insights using a dichotomy between the distal and the proximal pathways from environmental change to human health and wellbeing, recognizing the inherent complexity of most interactions. Macro and micro level processes continually interact and are tele-connected through systemic environmental processes, through the flows of material and mobility of populations around the world, and, importantly, through market and economic linkages [~~26~~44].

Climate change and distal food security

Food, nutrition and agricultural trade are potentially sensitive to climatic changes. [1,~~29~~47]. Rising levels of CO_2 both lead to a changing climate and can reduce the nutritional quality of crop production [~~30~~48]. Relative to an unchanging climate, yields of principal agricultural crops are already being affected globally, and have the potential to decline without major adaptations in technology and water use efficiency [1,~~29~~47,~~31~~49]. These changes are spatially sensitive, with risks of yield decrease likely to be greater in the hotter parts of the world [~~32~~50]. However, there is considerable scope for the food and trade-system to adapt to climate change [~~31~~49].

Comment [s49]: agree

This might be achieved via changes in the area of production (expansion of which may exacerbate climate change by liberating carbon in land conversion), and through impacts on trade and prices. However, given the complexity of the system, it is not clear how the multiple potential drivers of food availability and price will interact around issues such as: food for feed [~~33~~51]; biofuels [~~34~~52]; carbon pricing [~~35~~53]; water availability [~~36~~54]; competition for land and other resources [~~37~~55]; and the need for agriculture to be sustainable [~~38~~56]. Increasing weather variability may lead to short term unexpected shocks to supply [1,~~29~~47] that create significant volatility in food prices, impacting on the wellbeing of food insecure populations in all parts of the world.

Comment [s50]: agree – well written

Variation in prices driven by weather-related impacts have accentuated ~~accentuating~~ price shocks and created ~~-~~localized food shortages [~~39~~57]; both factors impact most, the poor sections of populations. ~~has its greatest impact on poor sections of populations.~~ For example, in the UK, a~~A~~nalysis of purchases following the 2007/8 global commodity food price spike show that as prices increased, households ~~in the UK, for example,~~ purchased 4.2% less food [~~40~~58] and bought lower quality alternatives. The greatest impact was on the poorest income decile: they spent 17% more in 2011 compared to 2007, so their relative food bill increased by 40% more than the UK average. On a global basis, food price spikes, driven by weather in the main bread-basket regions, directly impact market prices in the import-dependent low income countries, as well as indirectly influencing the food aid donated by the rich world. As a result, in sub-Saharan Africa particularly, the number of hungry increased following the 2007/8 and 2010/11 food price spikes. Furthermore, ~~Similarly,~~ the food price spike of 2010/11 has been estimated to have pushed >40

million people globally below basic needs poverty line in those years [~~41~~59]. Thus, weather impacts from climate change are likely to impact nutritional and future health status in all parts of the world and among consumers as well as producers [~~38~~56].

While food prices provide a proximal link between food security and climate change, the distal implications of climate change are profound. The growth of demand for food is driven by rising population size and wealth, and the need for sustainabilit~~l~~y. In many analyses demand is regarded as exogenous, driven by relationships with increasing wealth [~~42~~60], to which interventions need to be directed. However, the relationship between food and health are likely to shape trends in demand [~~33~~51,~~42~~60], and thus affect global agricultural production.

Climate change proximal and distal implications through migration and mobility

Climate changes involve spatial changes to economic and environmental systems that will prompt proximal and distal demographic responses. Fundamentally climate change will have an impact on where people live and on the decisions they make about moving from one location to another. Migration is a central element of economic and demographic change everywhere in the world. In effect, migration flows at the aggregate level are driven principally by differences in economic activity across space and time, though all individual decisions involve social, cultural and demographic dimensions. Some elements of the relative attractiveness of different areas, and hence the demand for migration, are sensitive to weather and climate. Hence resource scarcity, the availability of ecosystem services, and issues of

Comment [s51]: this may be out of date – see FAO revisions, eg see Revised hunger estimates accelerate apparent progress towards the MDG hunger target. *Global Food Security* (2015) 5(19-24.

Comment [GM52]: We have incorporated changes to reflect on this aspect.

Comment [CB53]: My understanding is that FAO, around 2014, revised their data, especially for this period – however, I accept that this statement is supported by this reference 59

security and hazard, all factor in the relative attractiveness of places and decisions to move between them [61-64~~43-46~~].

Comment [s54]: Also a good par

Climate changes have proximal and distal impacts on different types of migration. Displacement of populations from their place of residence as a result of extreme events is ~~usually~~ most often temporary and undertaken involuntarily, but has major public health and policy consequences. In the UK, for example, flood events temporarily displace people from their homes, often for months after events [~~47~~65]. The impacts of Hurricanes Katrina and Rita in Louisiana and New Orleans in 2005 showed that ~~temporary~~ displacement of populations from the flood impacts lead to very divergent ~~leads to highly differential permanent migration~~ patterns of who returned and who permanently migrated: ~~, with only~~ wealthier populations predominantly returned while poorer populations more frequently moved away permemantly~~ing~~, thus changing the demographics of the whole region in the long term [~~48~~66].

Comment [s55]: Maybe "can be temp" or "often temp" – because sometimes it is much more prolonged, as you say in the next par.

Comment [RS56]: We have amended this sentence as suggested by the reviewer.

Comment [RS57]: We have rephrased this section to make it more accessible – as indicated by the reviewer, our reference to differential migration patterns was not fully clear in the original version.

Climate change-induced resource scarcity reduces the potential for capital accumulation in resource-sensitive economies, and thus has a potential negative impact on the mobility potential of sections of the population who do not have the resources necessary for ~~migration~~ migration ~~prospects for migration~~. Hence, populations may experience a poverty-immobility nexus, ~~–~~ where increased mobility would be necessary for effective adaptation. In addition, ~~rapid urbanization, partly amplified by~~ migration trends of populations moving into expanding cities throughout developing and emerging economies~~,~~ means that a growing number of populations

Comment [s58]: Maybe for some - but from Syria, many have migrated despite poverty

Comment [RS59]: We agree with the reviewer comment and have rephrased this section.

are more exposed to weather and climate hazards in those migration destination areas.

A further interaction between migration and climate change is forced migration due to conflict. This type of migration is also ~~typically~~ involuntary, and has implications in both conflict areas and population-receiving areas. ~~However,~~ The direct links between climate risks and conflict risks ~~are~~ are not well established, yet the issue of attribution and causation is not the most relevant issue~~yet still an area of concern~~ [~~49~~67,~~50~~68]. The IPCC Fifth Assessment Report concludes that climate change impacts are likely to exacerbate poverty in resource-sensitive regions and that since poverty is a principal driver and predicator of violent conflict, the risks of climate change amplifying conflict risks in future are real [~~49~~67]. Conflict itself has significantly differential effects on the ability of populations to relocate from conflict zones [~~51~~70]. ~~The IPCC Fifth Assessment [1] emphasizes that C~~climate change, if it is to affect conflict risk, does so through expanding poverty as a principal cause of insecurity and conflict. Hence, in theory, there is a plausible route for increased risk in conflict-prone areas of the world over the incoming decades, in the absence of efforts for development and relief of the underlying causes of conflict in those regions [~~49~~67, 68].

The principal form of migration globally, however, continues to be the movement of populations to urban ~~centres~~centers within their national borders. In terms of absolute numbers, this trend is apparent and stark in Asia and Africa in particular [~~52~~71,~~53~~72]. Geographically, these migration trends are fueling trends of population movement towards coasts, and movement away from dry land and mountain environments [~~54~~73]. This dominant migration trend, in terms of numbers, creates significant environmental and public health challenges. The~~Hence on a global scale,~~

Comment [s60]: I think you mean not widely accepted, especially within the pol sci literature; suggest you be more explicit Kelley et al and Gleick (among others) would argue the evidence is strong as do some in the military.
Gleick P: Water, drought, climate change, and conflict in syria. *Weather, Climate, and Society* (2014) 6(331-340.
Kelley CP, Mohtadi S, Cane MA, Seager R, Kushnir Y: Climate change in the fertile crescent and implications of the recent syrian drought. *Proceedings of the National Academy of Sciences (USA)* (2015) 112(11):3241-3246.

Comment [RS61]: NEIL ADGER - Kelley et al often quoted as proof of link between drought and conflict in Syria. But this is a misreading. Kelley et al make no such claim – they only show how climate change exacerbates drought in the region. No analysis of climate conflict links. There is a lot of heat and no real evidence on the Syria case.

We have rephrased this section to reflect this comment and added further references to underpin this point.

Comment [CB62]: I disagree; this is not the place for debate, but it seems to depend on one's neural wiring! (ie to see the links or not) (I have corresponded extensively with Peter Geick re this.)

Comment [s63]: Would be good to cite Bowles et al, Climate change, conflict, and health. *Journal of the Royal Society of Medicine* (2015) **108**(10):390-395. AND perhaps mention these concerns are also long raised in health though little publicised (eg Lancet editorial 1989)

Comment [RS64]: Agreed, we have included this reference.

the movement of migrant populations into cities are differentially exposed to and the potential for climate hazards in those places: low income migrant communities are often located in flood prone zones or areas susceptible to landslides. Migrant populations cluster in areas with lowhigh density coastal mega-cities, with air quality and other affects, creates significant public health challenges not least for the migrants themselves or in slums with lack of access to sanitation or clean water [4631,4563]. Hence migration trends exacerbate environmental health risks: as many people are moving towards risks as moving away from them. These processes have both distal and proximal dimensions.

Conclusions

A weight of evidence suggests that climate-related environmental change in one part of the world will have systemic health and wellbeing impacts elsewhere at some point. Complex global interconnectivities underpin the pathways which are spatially and temporally distal. Vulnerability to health effects in geographically distant places is translated to individuals and communities by economic, social, ecological, biogeochemical, and resource flow mechanisms.

Future policies and interventions to deal with these risks need to account for how those risks are spatially and socially differentiated, and how their accessibility is dependent on a range of social -and cultural contexts, such that the benefits of those interventions are widespread [2,1020]. Similarly, the mitigation of climate change through decarbonisation of energy and altered economic systems have the potential to bring about significant benefits to health and wellbeing, especially if these are

widely distributed. Despite sentinel attempts over time by various commentators [see for example [14-17], there is still a need to ~~Concepts of public health that recognis~~help people in specific locations or countries (including policymakers) ~~e~~to understand and communicate ~~-~~climate-related health threats on vastly expanded temporal and spatial scales~~s~~. ~~, and the complexity of both distal and proximal causes would, we argue, help to realize such benefits.~~

The concept of ecosystem services and recent representations of their links to human health and wellbeing [~~19~~34,~~24~~6] demonstrate important links in many chains of causation. Both the benefits and dis-benefits of globalisation are unevenly distributed between and within countries and regions, and are invariably socially patterned and stratified to impact the most deprived. The complexity of proximal and distal ~~impacts,~~ pathways, suggests the need for a set of rapidly evolving novel qualitative and quantitative evidence and analysis techniques associated with the growth of big data in environment and human health research [74]~~.~~ The linking of ecosystem services to human health and wellbeing can be an important component in operationalising a new truly ecological public health. Communicating to a wide and diverse audience, that fostering better human health and wellbeing depends upon, and is intimately linked to, the changing state and sustainability of the Earth's geochemical and ecological systems, remains one of the greatest challenges of our time.

Comment [s65]: Important point but not original – should cite earlier work making exactly this point. More useful if you could comment why this point is so under-stressed

Comment [RS66]: We agree with the point made, but it would be beyond the scope of this paper to speculate based on current evidence and knowledge.

Comment [CB67]: OK

REFERENCES

1. International Panel on Climate Change. Working Group II contribution to the IPCC fifth assessment report climate change 2013: impacts adaptation and vulnerability summary for policymakers. 2014 http://www.ipcc-wg2.gov/AR5/. Last Access 16 October 2016.

2. Thomas F, Sabel CE, Morton K, Hiscock R, Depledge MH. Extended impacts of climate change on health and wellbeing Environ. Sci. Policy; 44:271–278, http://dx.doi.org/10.1016/j.envsci.2014.08.011.

3. Rayner G, Lang T. Ecological Public Health: Reshaping the Conditions for Good Health. Routledge Publishers, 2012.

4. McMichael AJ (1999) Prisoners of the proximate: loosening the constraints on epidemiology in an age of change. Am J Epidemiol. 15, 149(10):887-897.

5. Krieger N (2008) Proximal, Distal, and the Politics of Causation: What's Level Got to Do With It? American Journal of Public Health. 98: 221–30. doi: 10.2105/AJPH.2007.111278.

6. Millennium Ecosystem Assessment. Ecosystems and Human Wellbeing: Synthesis. Island Press, Washington; 2005.

7. Scottish Government (2008). Good Places Better Health: A New Approach to Environment and Health in Scotland – Implementation Plan. http://www.scotland.gov.uk/Publications/2008/12/11090318/0. Last Access 16 October 2016.

8. Scottish Government (2011). Good Places Better health- Supporting Documentation. http://www.scotland.gov.uk/Topics/Health/Healthy-Living/Good-Places-Better-Health. Last Access 16 October 2016.

9. NHS Health Scotland. A place standard for Scotland http://www.healthscotland.com/resources/cpps/local/placestandard.aspx, Last Access 16 October 2016.

10. Haines A, Fuchs C (1991) Potential impacts on health of atmospheric change. J Publ Health Med. 13:69-80.

11. Chivian E (1993) Critical Condition: Human Health and the Environment. MIT Press.

Comment [CB68]: Inconsistency in journal names, some shortened, others not

12. McMichael AJ (1993) Global environmental change and human population health: a conceptual and scientific challenge for epidemiology. Int J Epidemiol, 22:1-8.
13. Kovats RS, Haines A, Stanwell-Smith R, Martens P, Menne B, Bertollini R (1999) Climate change and human health in Europe. BMJ 318(7199):1682–5.
14. McMichael AJ, Haines A, Slooff R, Kovats S (1996) (Eds): Climate change and human health. World Health Organization, Geneva.
15. Butler CD, Corvalán CF and Koren HS (2005): Human health and well-being in global ecological scenarios. Ecosystems 8(2): 153-64.
16. Butler CD, Harley D (2010) Primary, secondary and tertiary effects of eco-climatic change: the medical response. Postgraduate Medical Journal 86:230-234.
17. Butler CD (2014) Climate Change and Global Health: a new conceptual framework. CAB Reviews: Perspectives in Agriculture, Veterinary Science, Nutrition and Natural Resources 9(27) doi: 10.1079/PAVSNNR20149027
18. Smith KR, Woodward A, Campbell-Lendrum D, Chadee DD, Honda Y, Liu Q, et al. Human health: impacts, adaptation, and co-benefits (2014). In: Climate Change 2014: Impacts, Adaptation, and Vulnerability. Part A: Global and Sectoral Aspects. Contribution of Working Group II to the Fifth Assessment Report of the Intergovernmental Panel on Climate Change, Cambridge University Press, Cambridge, United Kingdom and New York, NY, USA, 2014:709-754.
19. Department of Health (2001) 2001/2002 Health Effects of Climate Change in the UK, Crown Copyright.
20. Watts N, Adger WN, Agnolucci P, Blackstock J, Byass P, Cai W et al. (2015 Lancet Commission on Health and Climate Change: Policy Responses to Protect Public Health The Lancet, doi.org/10.1016/S0140-6736(15)60854-6
21. Lowe JA and Gregory JM (2005) The effects of climate change on storm surges around the United Kingdom. Phil. Trans. R. Soc. A 363:1313-1328, doi:10.1098/rsta.2005.1570.
22. Purvis MJ, Bates PD, Hayes CM (2008) A probabilistic methodology to estimate future coastal flood risk due to sea level rise. Coastal Engineering 55:1062–1073.
23. Thomas M, Pidgeon N, Whitmarsh L, Ballinger R (2015) Mental models of sea-level change: A mixed methods analysis on the Severn Estuary, UK. Global Environmental Change 33:71-82.

Comment [CB69]: spelling

24. Taylor AL, Dessai S, de Bruin WB (2014) Public perception of climate risk and adaptation in the UK: a review of the literature. Sustainability Research Institute Paper No. 63, SRI PAPERS, SRI Papers (Online) ISSN 1753-1330
25. Nurse LA, McLean RF, Agard J, Briguglio LP, Duvat-Magnan V, Pelesikoti N, Tompkins E, Webb A (2014) Small islands. In: Climate Change 2014: Impacts, Adaptation, and Vulnerability. Part B: Regional Aspects. Contribution of Working Group II to the Fifth Assessment Report of the Intergovernmental Panel on Climate Change [Barros, V.R., C.B. Field, D.J. Dokken, M.D. Mastrandrea, K.J. Mach, T.E. Bilir, M. Chatterjee, K.L. Ebi, Y .O. Estrada, R.C. Genova, B. Girma, E.S. Kissel, A.N. Levy, S. MacCracken, P .R. Mastrandrea, and L.L. White (eds.)]. Cambridge University Press, Cambridge, United Kingdom and New York, NY , USA, pp. 1613-1654. https://www.ipcc.ch/pdf/assessment-report/ar5/wg2/WGIIAR5-Chap29_FINAL.pdf, Last Access 16 October 2016
26. Redshaw CH, Stahl-Timmins WM, Fleming LE, et al. (2013) Potential changes in disease patterns and pharmaceutical use in response to climate change. J Toxicol Environ Health B Crit Rev.16(5):285-320. doi:10.1080/10937404.2013.802265.
27. Oppenheimer M, Campos M, Warren R, Birkmann J, Luber G, O'Neill B et al. (2014) Emergent risks and key vulnerabilities. In: Climate Change 2014: Impacts, Adaptation, and Vulnerability. Part A: Global and Sectoral Aspects. Contribution of Working Group II to the Fifth Assessment Report of the Intergovernmental Panel on Climate Change. Cambridge University Press, Cambridge, United Kingdom and New York, NY, USA, 2014:1039-1099.
28. Kovats RS, Depledge M, Haines A, Fleming LE, Wilkinson P, Shonkoff SB, Scovronick N (2014) The health implications of fracking. The Lancet 383(9919):757-758.
29. Lang T and Rayner G (2012) Ecological public health: the 21st century's big idea? An essay by Tim Lang and Geof Rayner. BMJ 345:e5466.
30. Morris GP (2010) Ecological public health and climate change policy. Perspectives in Public Health130(1):34-40.
31.Dahlgren G, Whitehead M. (1992) Policies and strategies to promote social equity and health. Copenhagen. World Health Organisation.
32. Evans RG, Stoddard GL (1994) Producing Health, Consuming Health Care. Soc Sci Med. 31:1359

33. Morris GP, Beck SA, Hanlon P, et al. (2006) Getting strategic about the environment and health. Public Health. 120:889–907.
34. Reis S, Morris G, Fleming LE, Beck S, Taylor T, White M, Depledge MH, Steinle S, Sabel CE, Cowie H, Hurley F, Dick JMcP, Smith RI, Austen M (2015) Integrating Health & Environmental Impact Analysis. Public Health 129(10):1383–1389. http://dx.doi.org/10.1016/j.puhe.2013.07.006.
35. Corvalan C, Briggs D, Kjellstrom T. (1996) Development of environmental health indicators. In: Briggs D, Corvalan, Nurminen N (eds). Linkage methods for environmental health analysis: general guidelines. WHO Geneva, 19-53.
36. World Health Organisation (2004). Environmental health indicators for Europe: a pilot indicator based report. World Health Organiz~~s~~ation Regional Office for Europe. http://www.euro.who.int/__data/assets/pdf_file/0003/140925/E82938.pdf. Last Access 16 October 2016.
37. Fisher B, Turner RK, Morling P (2009) Defining and classifying ecosystem services for decision making. Ecol Econ.68(3):643-53.
38. De Groot RS, Wilson MA, Bouwmans RMJ (2002) A typology for the classification, description and valuation of ecosystem functions, goods and services. Ecol Econ 41:393-408.
39. Daily GC, Soderquist T, Aniyar S, Arrow K, Dasgupta P, Ehrlich PR, Folke C, Jansson AM, Jansson BO, Kautsky N, Levin S, Lubchenco J, Maler KG, David S, Starrett D, Tilman D, Walker B (2000) The value of nature and the nature of value. Science 289:395–396.
40. Daily GC (1997) (Ed.) Nature's Services: Societal Dependence on Natural Ecosystems. Island Press, Washington, DC.
42. The UK National Ecosystem Assessment Technical Report (2011). UNEP-WCMC, Cambridge. http://uknea.unep-wcmc.org/. Last Access 16 October 2016.
43. Box GEP (1976) Science and Statistics. Journal of the American Statistical Association, 71:791–799, doi:10.1080/01621459.1976.10480949.
44. Adger WN, Eakin H, Winkels A. Nested and teleconnected vulnerabilities to environmental change. Front Ecol Environ (2009) 7(3):150–7, doi:10.1890/070148.
45. Liu J, Hull V, Batistella M, DeFries R, Dietz T, Fu F et al (2013) Framing sustainability in a telecoupled world. Ecology and Society 18(2):26.
46. Liu J, Mooney H, Hull V, Davis SJ, Gaskell J, Hertel T et al. (2015) Systems integration for global sustainability. Science. 347(6225):1258832

Comment [CB70]: an example of inconsistency in ref styles (eg compare with Ref 34)

Comment [CB71]: ?

47. Wheeler T and von Braun J (2013) Climate change impacts on global food security. Science.341(6145): 508-513.
48. Myers SS, Zanobetti A, Kloog I, Huybers P, Leakey AD, Bloom AJ et al. (2014) Increasing CO_2 threatens human nutrition. Nature.510:139-142.
49. Wheeler T and Reynolds C (2013) Predicting the risks from climate change to forage and crop production for animal feed. Animal Frontiers. 3(1):36-41.
50. Nelson GC, Valin H, Sands RD, Havlik P, Ahammad H, Deryng D, et al. (2013) Climate change effects on agriculture: Economic responses to biophysical shocks. PNAS, 111(9):3274-9.
51. Taheripour F, Hertel TW, Liu J (2013) The role of irrigation in determining the global land use impacts of biofuels. Energy, Sustainability and Society.3(1):1-18.
52. Smith P, Haberl H, Popp A, Erb K-H, Lauk C, Harper R, et al. (2013) How much land-based greenhouse gas mitigation can be achieved without compromising food security and environmental goals? Global Change Biology.19(8):2285-2302.
53. Immerzeel WW, van Beek LPH, Bierkens MFP (2010) Climate change will affect the Asian water. Science.328(5984):1382-5.towers.
54. Smith P, Gregory PJ, van Vuuren D, Obersteiner M, Havlik P, Rounsevell M, et al. (2012) Competition for land. Phil Trans Royal Soc Biol Sci.365(1554):2941-57.
55. Tscharntke T, Clough Y, Wanger TC, Jackson L, Motzke I, Perfecto I, et al. (2012) Global food security, biodiversity conservation and the future of agricultural intensification. Biological Conservation.151(1):53-59.
56. Browne JA, Hood A, Joyce R (2012) Child and Work-Age Poverty in Northern Ireland from 2010-2020. Institute of Fiscal Studies Report. http://www.ifs.org.uk/comms/r78.pdf. Last Access 16 October 2016.
57. Spratt S. (2013) Food price volatility and financial speculation. Future Agricultures Working Paper 47; Department for International Development. http://r4d.dfid.gov.uk/Output/192126/. Last Access 16 October 2016.
58. Dept for Environment, Food and Rural Affairs (DEFRA) (2012). Food Statistics Pocketbook. DEFRA, https://www.gov.uk/government/statistics/food-statistics-pocketbook-2012. Last Access 16 October 2016.
59. Ivanic M, Martin W, Zaman H. (2012) Estimating the short-run poverty impacts of the 2010-11 surge in food prices. World Development.40(11):2302-2317.

60. Valin H, Sands RD, van den Mensbrugghe D, Nelson GC, Ahammad H, Blanc E, et al. (2014) The future of food demand: understanding differences in global economic models. Agricultural Economics.45(1):51-67.

61. Black R, Bennett SR, Thomas SM, Beddington JR (2011) Climate change: Migration as adaptation. Nature 478(7370):447-449.

62. Piguet É, Pécoud A, de Guchteneire P (2011) (Eds.) Migration and Climate Change.Cambridge University Press, Cambridge; 2011.

63. Black R, Adger WN, Arnell NW, et al. (2011) The effect of environmental change on human migration. Glob Environ Change. 21(Suppl 1):S3–S11.

64. Geddes A, Adger WN, Arnell NW, et al. (2012) Migration, environmental change, and the challenges of governance. Environ Plann C Gov Policy.30(6):951-67.

65. Milojevic A, Kovats S, Leonardi G, et al. (2014) Population displacement after the 2007 floods in Kingston-upon-Hull, England. J Flood Risk Mgt. 9(2):99-104, doi.org/10.1111/jfr3.12111.

66. Fussell E, Sastry N, Van Landingham M (2010) Race, socioeconomic status, and return migration to New Orleans after Hurricane Katrina. Popul Environ 31:20–42.

67. Adger WN, Pulhin J, Barnett J et al. Human Security.(2014) In: Field C et al. (eds) IPCC Working Group 2: Impacts, Adaptation and Vulnerability. Cambridge 68. University Press 2014, http://www.ipcc.ch/report/ar5/wg2/. Last Access 16 October 2016.

68. Bowles et al. (2015), Climate change, conflict, and health. Journal of the Royal Society of Medicine 108(10):390-395.

69. Gleditsch NP (2012). Whither the weather? Climate change and conflict. Journal of Peace Research.49(1): 3-9.

70. Raleigh C (2011) The search for safety: the effects of conflict, poverty and ecological influences on migration in the developing world. Glob Environ Change 21(Suppl 1):S82–S93.

71. Parnell S, Walawege R. (2011) Sub-Saharan African urbanisation and global environmental change. Glob Environ Change. 21(Suppl 1):S12–S20.

72. Seto KC (2011) Exploring the dynamics of migration to mega-delta cities in Asia and Africa: contemporary drivers and future scenarios. Global Environ Change 21(Suppl 1):S94–S107.

73. deShernbinin A, Levy M, Adamo S, et al. (2012) Migration and risk: net migration in marginal ecosystems and hazardous areas. Environ Res Lett 7(4):045602.

1. 74. Fleming LE, Haines A, Golding B, Kessel A, Cichowska A, Sabel CE, Depledge MH, Sarran C, Osborne NJ, Whitmore C, Cocksedge N, Bloomfield D (2014) Data mashups: potential contribution to decision support on climate change and health. Int J Environ Res Public Health. 2014 Feb 4;11(2):1725-46. doi: 10.3390/ijerph110201725. International Panel on Climate Change. Working Group II contribution to the IPCC fifth assessment report climate change 2013: impacts adaptation and vulnerability summary for policymakers. 2014 http://www.ipcc-wg2.gov/AR5/. Accessed 22nd June 2015.
2. Thomas F, Sabel CE, Morton K, Hiscock R, Depledge MH. Extended impacts of climate change on health and wellbeing Environ. Sci. Policy; 2014, http://dx.doi.org/10.1016/j.envsci.2014.08.011.
3. Rayner G, Lang T. Ecological Public Health: Reshaping the Conditions for Good Health. Routledge Publishers, 2012.
4. Haines A, Fuchs C. Potential impacts on health of atmospheric change. J Publ Health Med. 1991;13:69-80
5. Chivian E, Critical Condition: Human Health and the Environment. MIT Press; 1993.
6. McMichael AJ. Global environmental change and human population health: a conceptual and scientific challenge for epidemiology. Int J Epidemiol. 1993;22:1-8.
7. Kovats RS, Haines A, Stanwell-Smith R, Martens P, Menne B, Bertolinni R. Climate change and human health in Europe. BMJ. 1999;318(7199):1682–5.
8. Department of Health 2001/2002 Health Effects of Climate Change in the UK, Crown Copyright.

9. Smith KR, Woodward A, Campbell-Lendrum D, Chadee DD, Honda Y, Liu Q, et al. Human health: impacts, adaptation, and co-benefits. In: Climate Change 2014: Impacts, Adaptation, and Vulnerability. Part A: Global and Sectoral Aspects. Contribution of Working Group II to the Fifth Assessment Report of the Intergovernmental Panel on Climate Change, Cambridge University Press, Cambridge, United Kingdom and New York, NY, USA, 2014:709-754.
10. Watts N, Adger WN, Agnolucci P, Blackstock J, Byass P, Cai W et al. Lancet Commission on Health and Climate Change: Policy Responses to Protect Public Health The Lancet. 2015 doi.org/10.1016/S0140-6736(15)60854-6
11. Redshaw CH, Stahl-Timmins WM, Fleming LE, et al. Potential changes in disease patterns and pharmaceutical use in response to climate change. J Toxicol Environ Health B Crit Rev. 2013;16(5):285-320. doi:10.1080/10937404.2013.802265.
12. Oppenheimer M, Campos M, Warren R, Birkmann J, Luber G, O'Neill B et al. 2014: Emergent risks and key vulnerabilities. In: Climate Change 2014: Impacts, Adaptation, and Vulnerability. Part A: Global and Sectoral Aspects. Contribution of Working Group II to the Fifth Assessment Report of the Intergovernmental Panel on Climate Change. Cambridge University Press, Cambridge, United Kingdom and New York, NY, USA, 2014:1039-1099.
13. Kovats RS, Depledge M, Haines A, Fleming LE, Wilkinson P, Shonkoff SB, Scovronick N. The health implications of fracking. Lancet 2014;383(9919):757 – 758.
14. Lang T, Rayner G. Ecological public health: the 21st century's big idea? An essay by Tim Lang and Geof Rayner. BMJ. 2012; 345: e5466.

15. Morris GP, (2010) Ecological public health and climate change policy. Perspectives in Public Health.130 No1 34-40.
16. Dahlgren G, Whitehead M. Policies and strategies to promote social equity and health. Copenhagen. World Health Organisation. 1992.
17. Evans RG, Stoddard GL. Producing Health, Consuming Health Care. Soc Sci Med. 1994;31:1359
18. Morris GP, Beck SA, Hanlon P, et al. Getting strategic about the environment and health. Public Health. 2006;120:889–907.
19. Reis S, Morris G, Fleming LE, et al. Integrating Health & Environmental Impact Analysis. Public Health. 2013. (in press). doi:10.1016/j.puhe.2013.07.006.
20. Corvalan C, Briggs D, Kjellstrom T. Development of environmental health indicators. In: Briggs D, Corvalan, Nurminen N (eds). Linkage methods for environmental health analysis: general guidelines. WHO Geneva; 1996:19-53.
21. World Health Organisation. Environmental health indicators for Europe: a pilot indicator based report. World Health Organisation Regional Office for Europe. 2004. www.euro.who.int/__data/assets/pdf_file/0003/140925/E82938.pdf. Accessed 22nd June 2015
22. Scottish Government. Good Places Better Health: A New Approach to Environment and Health in Scotland – Implementation Plan. 2008, http://www.scotland.gov.uk/Publications/2008/12/11090318/0. Accessed 22nd June 2015.
23. Scottish Government. Good Places Better health- Supporting Documentation. Scottish 2011 http://www.scotland.gov.uk/Topics/Health/Healthy-Living/Good-Places-Better-Health. Accessed 22nd June 2015.

24. Millennium Ecosystem Assessment. Ecosystems and Human Wellbeing: Synthesis. Island Press, Washington; 2005.
25. Fisher B, Turner RK, Morling P. Defining and classifying ecosystem services for decision making. Ecol Econ. 2009;68(3):643-53.
26. Adger WN, Eakin H, Winkels A. Nested and teleconnected vulnerabilities to environmental change. Front Ecol Environ 2009;7(3):150–7, doi:10.1890/070148.
27. Liu J, Hull V, Batistella M, DeFries R, Dietz T, Fu F et al Framing sustainability in a telecoupled world. Ecology and Society. 2013;18(2): 26. http://dx.doi.org/10.5751/
28. Liu J, Mooney H, Hull V, Davis SJ, Gaskell J, Hertel T et al. Systems integration for global sustainability. Science. 2015;347(6225):1258832
29. Wheeler T, von Braun J. Climate change impacts on global food security. Science. 2013a;341(6145): 508-13.
30. Myers SS, Zanobetti A, Kloog I, Huybers P, Leakey AD, Bloom AJ et al. Increasing CO2 threatens human nutrition. Nature. 2014;510: 139-142.
31. Wheeler T, Reynolds C. Predicting the risks from climate change to forage and crop production for animal feed. Animal Frontiers. 2013b;3(1):36-41.
32. Nelson, G C., Valin H, Sands RD, Havlik P, Ahammad H Deryng D et al. Climate change effects on agriculture: Economic responses to biophysical shocks. PNAS. 2013; 111(9):3274-9.
33. Taheripour F, Hertel TW, Liu J. The role of irrigation in determining the global land use impacts of biofuels. Energy, Sustainability and Society. 2013;3(1):1-18.
34. Smith P, H. Haberl H, Popp A, Erb K-h, Lauk C, Harper R, et al. How much land-based greenhouse gas mitigation can be achieved without compromising food

security and environmental goals? Global Change Biology. 2013; 19(8):2285-2302.

35. Immerzeel WW, van Beek LPH, Bierkens MFP. Climate change will affect the Asian water. Science. 2010;328(5984):1382-5.towers.
36. Smith P, Gregory PJ, van Vuuren D, Obersteiner M, Havlik P, Rounsevell M, et al. Competition for land. Phil Trans Royal Soc Biol Sci. 2010;365(1554):2941-57.
37. Tscharntke T, Clough Y, Wanger TC, Jackson L, Motzke I, Perfecto I, et al. Global food security, biodiversity conservation and the future of agricultural intensification. Biological Conservation. 2012;151(1):53-9.
38. Browne JA, Hood A, Joyce R. Child and Work-Age Poverty in Northern Ireland from 2010-2020. Institute of Fiscal Studies Report. 2012. http://www.ifs.org.uk/comms/r78.pdf. Accessed 22nd Feb 2015.
39. Spratt S. Food price volatility and financial speculation. Future Agricultures Working Paper 47; Department for International Development. 2013. http://r4d.dfid.gov.uk/Output/192126/. Accessed 22nd June 2015.
40. 40 Dept for Environment, Food and Rural Affairs (DEFRA). Food Statistics Pocketbook. DEFRA, 2012 Available at https://www.gov.uk/government/statistics/food-statistics-pocketbook-2012
41. Ivanic M, Martin W, Zaman H. Estimating the short-run poverty impacts of the 2010-11 surge in food prices. World Development. 2012;40(11):2302-2317.
42. Valin H, Sands RD, van den Mensbrugghe D, Nelson GC, Ahammad H, Blanc E, et al. The future of food demand: understanding differences in global economic models. Agricultural Economics. 2014;45(1):51-67.
43. Black R, Bennett SR, Thomas SM, Beddington JR. Climate change: Migration as adaptation. Nature. 2011;478(7370): 447-449.

44. Piguet É, Pécoud A, de Guchteneire P, eds. Migration and Climate Change.Cambridge University Press, Cambridge; 2011.
45. Black R, Adger WN, Arnell NW, et al. The effect of environmental change on human migration. Glob Environ Change. 2011;21 Suppl 1:S3–S11.
46. Geddes A, Adger WN, Arnell NW, et al. Migration, environmental change, and the challenges of governance. Environ Plann C Gov Policy. 2012;30(6):951–67.
47. Milojevic A, Kovats S, Leonardi G, et al. Population displacement after the 2007 floods in Kingston-upon-Hull, England. J Flood Risk Mgt. 2014;in press. doi.org/10.1111/jfr3.12111.
48. Fussell E, Sastry N, Van Landingham M. Race, socioeconomic status, and return migration to New Orleans after Hurricane Katrina. Popul Environ. 2010;31:20–42.
49. Adger WN, Pulhin J, Barnett J et al. Human Security. In: Field C et al. (eds) IPCC Working Group 2: Impacts, Adaptation and Vulnerability. Cambridge University Press 2014, http://www.ipcc.ch/report/ar5/wg2/ Accessed 22nd June 2015
50. Gleditsch NP. (2012). Whither the weather? Climate change and conflict. Journal of Peace Research. 2012;49(1): 3-9.
51. Raleigh C. The search for safety: the effects of conflict, poverty and ecological influences on migration in the developing world. Glob Environ Change. 2011;21 Suppl 1.:S82–S93.
52. Parnell S, Walawege R. Sub-Saharan African urbanisation and global environmental change. Glob Environ Change. 2011;21 Suppl 1.:S12–S20.

53. Seto KC. Exploring the dynamics of migration to mega-delta cities in Asia and Africa: contemporary drivers and future scenarios. Global Environ Change. 2011;21 Suppl 1.:S94–S107.

54. deShernbinin A, Levy M, Adamo S, et al. Migration and risk: net migration in marginal ecosystems and hazardous areas. Environ Res Lett. 2012;7(4):045602.

Author response

Reviewer 1: Hilary Graham

This is an interesting and clearly-written paper addressing what many regard as the major 21st century threat to public health. It does not present new findings and perspectives; its primary concern, instead, is to bring together existing evidence and frameworks in ways that highlight the multiple pathways through which the health effects of climate change are mediated. As I read the Abstract, this task is undertaken in order to aid public health planning and the conceptualization of interventions to mitigate and adapt to changing environmental conditions.

There are three areas where the paper could be strengthened.

Firstly, I would recommend that a clearer statement of aims and methods is given in the Abstract and then in the body of the paper. Currently, the Abstract tells the reader what the paper does ('reviews evidence' 'presents a conceptual model') but does not explain why and to what end these exercises are being undertaken. A statement of aims would help here, and should also be included in the Introduction section of the paper. Relatedly, the Abstract provides no information on methods (how the evidence was accessed and assessed etc.) – and again the body of the paper has no discussion of the methods used to scope and review the evidence and scope and review potentially-relevant conceptual models. Food systems and migration feature as case studies in the later part of the paper; signally these foci in the Abstract and explaining them in the Introduction would again be helpful.

Related to this first point, it is not altogether clear where the originality of the paper lies. A crisper Abstract and Introduction would ensure that the reader is left in no doubt about this. To explain: with respect to the evidence review part of the paper, there are many existing reviews and reports summarizing evidence on health impacts of climate change and the complex pathways through which these impacts occur, including both those cited in the paper and others. So the reader may be asking 'what does the paper add?' With respect to the conceptual model, the one presented is taken from the authors' previously-published work – again the reader may therefore ask 'where is the originality?'

We have reinforced both the abstract and the introduction to better address this criticism. Specifically, we have alluded to the relevance of the work to national public health planning. We recognize the overlap of proximal pathways with the direct health effects and the overlap of Distal with indirect, secondary and tertiary effects described elsewhere but we would submit this is an overlap and not a direct alignment. In our world, proximal and distal are always defined with reference to a specific population and our paper is about the benefits to public health and policy in a particular location an appreciation of the proximal and distal pathways and how they track back to a population's own health. We have proposed that this appreciation can be greatly assisted by the product and process of populating conceptual models for a particular issues. The principle of using issue framing in

environmental health issues has been trialled to good effect in a Scottish environmental health policy context and can we submit be applied in an extended temporal and spatial scale. These insights are where we argue the originality of the paper lies.

Secondly, the concepts of 'proximal' and 'distal' anchor the paper. These are very familiar terms within social epidemiology and public health – and have been the subject of influential critiques. The authors should provide definitions of what they mean by the terms, particularly as they use them to refer to various points along the causal chain: proximal and distal determinants (and stressors and threats), proximal and distal pathways, proximal and distal experiences and proximal and distal effects. If they wish to discuss how they usage of the terms relates to and/or is informed by wider critiques of the terms, examples include Tony McMichael's 1999 paper 'Prisoners of the Proximate' and Nancy Krieger's 2008 paper 'Proximal, Distal, and the Politics of Causation'.

We recognize the importance of Tony McMichael's Prisoners of the Proximate and also of Nancy Kriegers papers in relation to the terms proximate and distal and have now referenced these papers. We very specifically use proximal and distal here in reference to climate change related pathways to human health for a specific country or location and we would respectfully submit they are sufficient defined in the paper.

Thirdly, there is relatively little attention given to what the evidence review and conceptual model mean in practice for public health planning and interventions (this was signaled as part of the paper's remit in the Abstract). I was expecting a section discussing how a focus on the 'proximal' and 'distal' domains helped with policy development and evaluation, particularly given the authors' important emphasis in the Introduction on equity and sustainability across societies and generations. I would suggest either this aspect of the paper is strengthened, or the Abstract and Introduction are revised to downplay it.

We appreciate the comments of the reviewer and while the relevance of the concepts we discuss for the policy domain is without any doubt an important aspect, we feel that it is too early to gauge any potential impact. The proximal and distal concept has been introduced into the discussion in the context of the UK Living With Environmental Change, with a focus on health. Our examples and other literature indicate a growing awareness and take-up of ecological public health and systematic/integrated thinking in the policy and planning process. However, adequately reflecting on how these concepts (will) support policy and planning will require both more time to analyse outcomes, but as well a wider discussion including all relevant actors within a transdisciplinary framework, which goes beyond the remit and scope of our current paper. We suggest that this point is best addressed by a follow-up publication down the line, which can address this with suitable depth and based on emerging evidence from the policy domain.

In addition to these three areas, I would encourage the authors to address two other points. First, the paper introduces an ecosystem focus (2nd para of the Introduction). Does the term

need defining and this focus explaining – and then drawn more clearly through the rest of the paper?

We refer to the ecosystem focus as it has been introduced by Rayner and Lang in their Ecological Public Health works, and link this to the conceptual understanding of how ecosystems and health are linked based on the MEA, the UK National Ecosystem Assessment and a body of peer-reviewed literature around those. We believe that a further definition beyond these foundations is not adding value to the paper, but have made several revisions/edits to the body of the text in response to this and the other reviewer's comments, which we are confident will improve the communication of our conceptual understanding of the ecosystem focus.

Secondly, while references are cited to support many statements in the paper, there are a number of fairly bold statements that have no evidence cited in their support.

We accept this criticism, which has as well been reflected by the comments of the next reviewer. As a consequence, we have added a substantial number of new and additional references, making relevant additions throughout the text where our original manuscript had not provided sufficiently robust underpinning of the claims and statements made.

Reviewer 2

Thanks for the chance to comment on this paper, "Scoping the Proximal and Distal Dimensions of Climate Change on Health and Wellbeing".

There was much within it with which I generally agree, but the antecedents of many of the concepts here should be better acknowledged.

There is substantial conceptual overlap, with earlier work, in particular see:

Butler C.D., Corvalán, C.F. and Koren, H.S. (2005): Human health and well-being in global ecological scenarios. *Ecosystems* **8**(2): 153-64.

Butler C.D., Harley D. (2010) Primary, secondary and tertiary effects of eco-climatic change: the medical response. *Postgraduate Medical Journal* 86:230-234.

Butler C.D. (2014) Climate Change and Global Health: a new conceptual framework CAB Reviews: *Perspectives in Agriculture, Veterinary Science, Nutrition and Natural Resources* 9(27) doi: 10.1079/PAVSNNR20149027

I know I am the first author of these three (all peer reviewed) and it perhaps seems brash to highlight my own work, but there is much overlap and I really think the lead author should read all three and cite at least one. There is also an edited book, building on the conceptual framework (Butler C.D., (2016), Climate Change and Global Health. CABI, Wallingford, UK, 318+xxiv pages (softcover edition)). Approximately 1/5th of this book discusses content very similar to what you have called "distal" – I called it "tertiary".

Otherwise, the readers of the final piece may not recognise this overlap and thus overestimate its conceptual originality. It is perhaps also worth mentioning that the final section of the health chapter in the recent IPCC report made a similar statement about what you call distal effects, though, unfortunately, without providing a citation.

We wholly accept and apologise for our failure to reference this work – this was an omission on our part. The omission was an error with origins in the evolution of the paper from a very UK centric, document to the paper it now is. We have introduced references to both McMichael et al and three papers in which the reviewer was lead author and rephrased/revised text accordingly.

I also think it would be worth stating that none of these conceptual models are complete, ideal or perfect .. rather, they are like different map projections (Mercator, Peters etc) with strengths and weaknesses. Ultimately the value of these mental models depends too on the mind of the reader; i.e. the existing concepts and thoughts with which readers of this paper build on.

We have now addressed this, albeit perhaps too concisely by introducing the quotation from George Box after the discussion of the model. I accept a fuller discussion might be helpful but conscious of space, we felt it useful to express a measure of realism about models.

We have, in addition to this response, provided a commented and track-changed version of the revised manuscript in order to allow the editor and reviewers to more readily assess the nature and degree of revisions made.
